# Conduct and reporting of citation searching in Cochrane systematic reviews: A cross‐sectional study

**DOI:** 10.1002/jrsm.1355

**Published:** 2019-07-04

**Authors:** Simon Briscoe, Alison Bethel, Morwenna Rogers

**Affiliations:** ^1^ Exeter HS&DR Evidence Synthesis Centre, College of Medicine and Health University of Exeter Exeter UK; ^2^ Evidence Synthesis Team, NIHR CLAHRC South West Peninsula, College of Medicine and Health University of Exeter Exeter UK

**Keywords:** checking references, citation searching, Cochrane reviews, literature searching

## Abstract

**Background:**

The search for studies for a systematic review should be conducted systematically and reported transparently to facilitate reproduction. This study aimed to report on the conduct and reporting of backward citation searching (ie, checking reference lists) and forward citation searching in a cross section of Cochrane reviews. Citation searching uses the citation network surrounding a source study to identify additional studies.

**Methods:**

Cochrane reviews were identified by searching the Cochrane Database of Systematic Reviews using the wildcard symbol and date limiting to the 3‐month period November 2016 to January 2017. Cochrane reviews thus identified were screened for mention of citation searching. Descriptive detail on the conduct and reporting of citation searching was captured in data extraction forms and described and evaluated.

**Results:**

Two hundred fifteen Cochrane reviews were identified. One hundred seventy‐two reviews reported backward citation searching, and 18 reviews reported forward citation searching. Web of Science was the most frequently reported citation index. The studies used for backward citation searching consisted mainly of studies meeting the inclusion criteria. One‐third of reviews that reported forward citation searching used selected studies of importance. Reporting of citation searching was compliant with the Methodological Expectations of Cochrane Intervention Reviews (MECIR) standards, but full transparency requires additional detail that only a minority of reviews reported.

**Conclusion:**

The conduct of backward citation searching was more uniform than forward citation searching. This might be due to lack of MECIR guidance for forward citation searching. Reporting was generally compliant with MECIR, but this is not always sufficient to ensure full transparency.

## 1 INTRODUCTION

Systematic reviews aim to answer research questions by identifying, appraising, and synthesizing all the relevant evidence.^1^ An important component of a systematic review is the search for studies, which aims to identify all studies that answer the research question. In conformity with the overall methodology for a systematic review, the search for studies should be conducted using systematic and reproducible methods and documented such that it can be reported transparently.^2^ This study reviews how two similar search methods, backward and forward citation searching (hereafter, citation searching, unless one or the other is explicitly stated), were conducted and reported in a cross section of Cochrane systematic reviews (hereafter, Cochrane reviews) published in a 3‐month period.

### 1.1 What is and why conduct citation searching?

Citation searching uses the citation network surrounding a source study to identify similar studies. A citation network consists of the studies that are cited by a source study (ie, the reference list) and the studies that cite a source study. Citation searching in the context of a systematic review usually starts with one or more studies that meet, or that have similar content to, the inclusion criteria. We use the term *study* herein synonymously with *article* to refer to a document that describes the methods and results of primary or secondary research. Potential candidate studies for citation searching include the following:

- Selected key studies of particular importance;
- All studies eligible for inclusion in a review;
- Potentially relevant studies, such as studies included at title and abstract screening for full‐text screening.^3^

On the assumption that studies that cite or are cited by a source study are likely to have similar content, the citation network is searched backward and/or forward: *Backward citation searching* involves inspecting the references that are cited in the source study (hence often called *checking reference lists*), and *forward citation searching* involves using a citation index to identify studies that cite a source study.^2^, ^4^

Citation indexes are bibliographic databases that index citations of studies in addition to the standard bibliographic content. They include Scopus (Elsevier, USA) and Web of Science (Clarivate Analytics, USA), which are both subscription‐based, and the freely available Google Scholar. Web of Science is composed of several subject specialist databases, access to which varies depending on the user's subscription. The Web of Science Core Collection includes the Science Citation Index, the Social Sciences Citation Index, the Arts & Humanities Citation Index, and conference proceedings.^5^ Google Scholar, Scopus, and Web of Science have similar but not identical journal coverage, which can result in variation in the number of citations identified for the same source study.^6^ For example, a forward citation search of Whear et al^7^ identifies 29 citations in Web of Science, 37 citations in Scopus, and 65 citations in Google Scholar (search conducted by S.B. on 1 November 2018). This phenomenon has raised the question, still to be resolved conclusively, of whether searching multiple citation indexes is preferable to one citation index^8^; reasons against this approach include time and resource implications. Backward citation searching can be conducted manually by inspecting the reference list of the source study or via Scopus or Web of Science, which both index reference lists of studies as well as citations of studies.

Citation searching typically supplements searching bibliographic databases when searching to identify studies for a systematic review.^9^ A cross‐sectional study of 300 systematic reviews found that 81% reported backward citation searching and 12% reported forward citation searching as an adjunct to searching one or more bibliographic database.^10^ The aim of citation searching is to identify studies missed by text‐based searches in the title, abstract, or controlled vocabulary fields of bibliographic records.^9^ Studies that compare the effectiveness of citation searching with searching bibliographic databases show that citation searching is particularly effective at retrieving studies for systematic reviews where core concepts are difficult to capture using keywords, eg, where core concepts are described inconsistently due to systemic reporting deficiencies, or due to historical development of terminology in a subject area or research methodology.^9^, ^11^, ^12^, ^13^, ^14^, ^15^ Iterative citation searching using studies identified by citation searching, or *citation snowballing*, might be useful for systematic reviews of hard‐to‐find studies, such as those included in qualitative evidence synthesis.^16^ In these types of review, citation searching can be considered a complementary or even primary search method rather than as supplementary to searching bibliographic databases.^15^, ^17^ Citation searching yields fewer unique studies where the search query can be successfully represented by a text‐based search.^18^ However, it can still be useful for identifying studies not indexed in the bibliographic databases searched, or identifying studies before they are indexed in a bibliographic database.^18^

### 1.2 Cochrane guidance on citation searching: summary and commentary

Guidance and methodological standards on searching for studies for Cochrane reviews are found in the “Searching for Studies” chapter of the Cochrane Handbook for Systematic Reviews of Interventions (hereafter, Cochrane Handbook)^2^ and the Methodological Expectations of Cochrane Intervention Reviews (hereafter, MECIR standards).^19^, ^20^ The Cochrane Handbook^2^ provides detailed guidance on searching for studies, and the MECIR standards describe the mandatory and desirable standards of conduct^20^ and reporting.^19^ At the time of writing, the Cochrane Handbook is undergoing revision in preparation for a new edition (version 6). The summary and commentary below on citation searching guidance refer to the currently available version (5.1), which is the version that the authors of the Cochrane reviews in our cross section have used to inform their search methods.^2^

The MECIR standards for *conducting* Cochrane reviews stipulate that backward citation searching is mandatory (C30) *
Numbers in parentheses in this section refer to the relevant MECIR standard item on either conduct (eg, C30) or reporting (eg, R33). alongside searching a core set of bibliographic databases (C24).^20^ In particular, review authors should use *included studies* and *any relevant systematic reviews* when conducting backward citation searching.^20^ There is no guidance in the MECIR standards^20^ or Cochrane Handbook^2^ on whether to use a manual or citation index‐assisted approach, leaving it open to the searcher to determine the most appropriate method. Although forward citation searching is not mentioned in the MECIR standards, the Cochrane Handbook recommends it as an important adjunct to searching bibliographic databases.^2^ Also in the Cochrane Handbook^2^ is the suggestion that an important relevant article might be a good starting point for forward citation searching, implying that a more focused approach may be taken than for backward citation searching (cf C30).^20^ No specific single or combination of citation indexes is recommended in the MECIR standards^20^ or Cochrane Handbook,^2^ leaving it open to the searcher to determine the most appropriate tool or tools. There is a warning in the Cochrane Handbook that, because citations are susceptible to biases such as selective citation of studies with positive results, citation searching is not an objective search method and the results should be used with caution.^2^

The MECIR standards for the *reporting* of search methods in Cochrane reviews stipulate that review authors should “[l]ist all sources searched, including … whether reference lists were searched” (R33).^19^ Although forward citation searching is not explicitly mentioned, any citation indexes used should be included in the list of sources searched. This also applies to backward citation searching in reviews where a citation index is used for this purpose.

This is the full extent of detail required by the MECIR standards to report about citation searching.^19^ In addition, we suggest that it is useful to report the set of studies used for citation searching. In particular, if the set of studies used is a narrower or broader set than the studies included in the review, then the specific studies should be listed, eg, key studies of interest or studies not included in the review. This allows the scope of the search to be assessed and facilitates reproduction. Furthermore, we suggest explicitly stating that a citation index was used for citation searching as citation indexes can also be searched using keywords. Finally, reporting the date of forward citation searching allows the timeliness of the search to be assessed. The date of the search is not relevant for backward citation searching as reference lists remain the same over time.

A summary of key methodological decisions required when conducting citation searching is presented in Table 1. The only mandatory requirement in the MECIR standards on conducting citation searching is that included studies are used for backward citation searching. A summary of the reporting guidance in the MECIR standards combined with our suggestions and commentary is described in Table 2.

**Table 1:** Key methodological decisions when conducting citation searching

	Methodological decision	Commentary
1	What set of studies to use	The minimum standard for BCS for Cochrane reviews is included studies and any relevant systematic reviews. A more selective approach can be used for FCS if appropriate.
2	What citation index to use and whether to use more than one citation index	The main options are Google Scholar, Scopus and Web of Science. Coverage varies between citation indexes.
3	Whether to use a manual or citation index‐assisted approach for BCS	A manual approach is perhaps the best way of ensuring all citations are checked as there is a risk that a citation index fails to index all cited studies. A benefit of using a citation index is the option to export and de‐duplicate the results of a large set of citations from multiple studies to avoid the potential for screening the same cited study or studies multiple times.
4	Whether to use a non‐standard approach	For example, citation snowballing. Can be particularly useful for identifying hard‐to‐find literature, such as qualitative studies.

Abbreviations: BCS, backward citation searching; FCS, forward citation searching.

**Table 2:** MECIR standards and suggested checklist for the reporting of citation searching

	Detail	MECIR		Commentary
		BCS	FCS	
1	Name of citation index(es) or manual approach	Yes if citation index is used (R33)	Yes (R33)	Provides the reader with important detail on what was done. This should include a statement that the citation index was used for citation searching in particular, as citation indexes can also be searched using keywords.
2	Set of studies used	No	No	To ensure transparency, wherever possible the specific studies used should be listed if other than all included studies.
3	Date of search	‐	No	Useful for FCS. Not required for BCS as the results do not change over time.

Abbreviations: BCS, backward citation searching; FCS, forward citation searching.

### 1.3 Rationale, aims, and objectives

We conducted this study because we wanted to better understand variations in the conduct and reporting of citation searching in systematic reviews. To date, reviews on the conduct and reporting of search methods in systematic reviews have reported findings on citation searching relatively briefly—typically, the prevalence of the search method.^10^, ^21^ There are several published case studies of citation searching^11^, ^12^, ^13^, ^14^, ^18^ and reviews of such studies,^3^, ^9^ but their focus is the effectiveness of the search method compared with other search methods (usually in a single case study) rather than a cross‐sectional analysis of conduct and reporting. We chose to examine Cochrane reviews in particular because they are a more consistently high standard of systematic review than other types of systematic review.^22^

We had two main aims. First, we aimed to describe how citation searching was conducted in a cross section of Cochrane reviews. This included five specific objectives on the conduct of citation searching derived from our experience as information specialists and the relevant literature (summarized in Table 1), namely, to describe the following:

1. The different sets of studies that were used for citation searching and how frequently;
2. The citation indexes that were used and how frequently;
3. The frequency of a manual approach for backward citation searching versus a citation index assisted approach;
4. The frequency of using more than one citation index for citation searching;
5. The frequency of citation snowballing and/or detection of other nonstandard approaches to citation searching.

Secondly, we aimed to assess whether the reporting of how citation searching was conducted was transparent and reproducible, in fulfilment of the minimum reporting standard required for a Cochrane review.^1^ This included the three items in Table 2 (ie, name of citation index, set of studies used, and the date of the search) and also where in the systematic review citation searching was reported.

## 2 METHODS

### 2.1 Eligibility criteria

We included Cochrane reviews (including both new and update reviews) if they used backward or forward citation searching to identify studies. It was not sufficient for inclusion that a review listed a citation index in the list of databases searched, as this could mean that the citation index was searched using a text‐based search strategy. Instead, we looked for explicit description that citation searching was conducted.

The publication date of included reviews was limited to the 3‐month period November 2016 to January 2017. This was due to practical constraints of time and resources that prevented looking at a larger cross section; and it was a convenience sample from an earlier review on the reporting of web searching in Cochrane reviews by S.B.^23^

Cochrane reviews that reported identifying no studies that met their inclusion criteria were excluded because these reviews had no or limited opportunity to conduct citation searching. Some such reviews reported an intention to conduct citation searching, but because we were interested in actual practice of citation searching, we did not include these reviews in our analysis. Cochrane reviews that were withdrawn from publication were also excluded due to potential shortcomings in the search methods that would not reflect acceptable practice when searching for studies for Cochrane reviews.

### 2.2 Identification of Cochrane reviews

Cochrane reviews from the 3‐month period November 2016 to January 2017 were identified by searching the Cochrane Database of Systematic Reviews via the Cochrane Library using the asterisk (ie, wildcard) symbol in the Search All Text search field and date‐limiting using the Online Publication Date feature. The results were then exported to Endnote X7 (Thomson Reuters, New Mexico, USA). This process was undertaken in February 2017 by S.B. as part of an earlier study on the reporting of web searching in Cochrane reviews.^23^ All Cochrane reviews in the Endnote library were downloaded and inspected for detail about citation searching by S.B. This involved manual inspection of the abstract, methods, and appendices of reviews for any mention of citation searching and using the Control‐F search feature to search for keywords and phrases such as *backward*, *forward*, *citation*, *reference list*, *Web of Science*, *Scopus*, and *Google*.

### 2.3 Data‐extraction and categorization

We developed a data‐extraction form to capture details about how citation searching was conducted and reported. The form was developed with reference to the MECIR standards^19^, ^20^ and our wider background reading and recommendations for good practice (see Tables 1 and 2). After a pilot run by all authors on a sub‐set of the sample, S.B. inspected all the included Cochrane reviews in the sample and data‐extracted key details relating to our five specific aims, including whether backward or forward citation searching was reported; what citation index was reported (or manual approach); the set of studies used to conduct citation searching; any additional details reported about citation searching; and where detail about citation searching was reported. M.R. data‐extracted a 10% subset of the sample that were cross‐checked with S.B.'s data‐extraction forms for consistency. Discrepancies were resolved through discussion after the data‐extraction process was complete.

We coded reviews that did not explicitly state whether a manual or citation index‐assisted approach was used for backward citation searching as *manual*. In our extensive collective experience of searching for studies for systematic reviews within several UK research institutions, researchers have almost always opted to conduct backward citation searching manually—hence, we considered this a reasonable assumption. However, we also acknowledge that this was only an assumption and kept a separate record of the number of reviews that explicitly reported using a manual approach.

We applied categories to describe the different sets of studies used for citation searching that we identified. These included the following: *key studies* (ie, studies selected as of outstanding importance for the review); *included studies* (ie, studies that met the inclusion criteria for the review, variously described in our sample as *included*, *eligible*, and *relevant* studies); and *identified studies* (ie, studies identified by other search methods that may or may not be relevant to the review, variously described in our sample as *identified*, *retrieved*, and *potentially relevant* studies). These categories are all mutually exclusive. However, we acknowledge that the intended meaning of *identified* is ambiguous and might have been used synonymously with *included* in some cases. As such, the distinction made by our categorization might in some cases be semantic rather than procedural.

We also had a category for *systematic reviews* that is not mutually exclusive, as review authors can conduct citation searching on both primary studies and systematic reviews.

## 3 RESULTS

### 3.1 Selection of Cochrane reviews

We identified 215 Cochrane reviews with online publication dates from November 2016 to January 2017. We excluded 17 reviews from all subsequent analysis, including seven reviews that were withdrawn from publication and 10 reviews that failed to identify any source studies for citation searching via the bibliographic database searches, ie, no studies that met the inclusion criteria for the review. Of the remaining 198 reviews, 172 (87%) reported backward citation searching, and 18 reviews (9%) reported forward citation searching. The 18 reviews that reported forward citation searching were published by 14 different Cochrane review groups (Airways^24^; Anesthesia^25^; Common Mental Disorders^26^; Developmental, Psychosocial, and Learning Problems^27^; Dementia and Cognitive Improvement^28^, ^29^; Effective Practice and Organization of Care^30^, ^31^; Eyes and Vision^32^; Heart^33^, ^34^; Injuries^35^; Musculoskeletal^36^; Neonatal^37^, ^38^; Stroke^39^; Vascular^40^; and Wounds^41^). No reviews reported forward citation searching without also reporting backward citation searching.

### 3.2 Conduct of citation searching

Here, we present our findings on how citation searching was conducted in the sample. Table 3 presents overall findings for each of our five objectives regarding the conduct of citation searching.

**Table 3:** Conduct of citation searching in cross‐section of Cochrane reviews (n = 198)

	Item of Conduct	Descriptive Detail	BCS	FCS
			n = 172	n = 18
1	Set of studies used^a^	**Named set of studies**	**159 (92)**	**15 (83)**
		Key studies	3 (2)	5 (33)
		*Key studies (reported)*	*0 (0)*	*2 (13)*
		*Key studies (not reported)*	*3 (2)*	*3 (20)*
		Included studies	94 (59)	8 (53)
		Identified studies	62 (39)	2 (13)
		*Identified studies (reported)*	*0 (0)*	*0 (0)*
		*Identified studies (not reported)*	*62 (39)*	*2 (13)*
		Systematic reviews	65 (41)	1 (7)
2	Citation index used	**Named citation index**	**0 (0)**	**15 (83)** b
		Google Scholar	‐	1 (7)
		Scopus	‐	2 (13)
		Web of Science	‐	13 (87)
		*Science Citation Index*	‐	*7 (47)*
		*Core Collection*	‐	*1 (7)*
3	Citation index/manual for BCS	**Citation index**	**0 (0)**	**‐**
		**Manual**	**172 (100)**	**‐**
		Manual (reported)	3 (2)	‐
		Manual (assumed)	169 (98)	‐
4	Citation indexes per review	1 citation index	‐	14 (93)
		2 citation indexes	‐	1 (7)
5	Non‐standard approaches	Snowballing	0 (0)	0 (0)
		Other	0 (0)	0 (0)

*Note*. Numbers outside parentheses are totals, and numbers inside parentheses are percentages. Percentages in bold are of the overall set of included reviews for backward (n = 172) and forward (n = 18) citation searching; all other percentages are of the subset of reviews for the relevant item of conduct; eg, 83% of reviews that reported forward citation searching reported the set of studies used, and 5% of this subset of reviews reported using key studies.

Abbreviations: BCS, backward citation searching; FCS, forward citation searching.

a Sets of studies (ie, key, included, and identified) are mutually exclusive except for systematic reviews.

b The total number of reviews that named a citation index for forward citation searching is less than the sum total of named citation indexes because a proportion of reviews named more than one citation index (see item of conduct 4).

#### 3.2.1 What sets of studies are used and how frequently?

Of the 172 Cochrane reviews that reported backward citation searching, 159 (92%) reported the set of studies that were used. Of the 18 reviews that reported forward citation searching, 15 (83%) reported the set of studies used.

*Key studies* were used for backward citation searching in three of 159 (2%) reviews that reported a set of studies and forward citation searching in five of 15 (33%) reviews that reported a set of studies. The specific studies on which citation searching was conducted were reported in two reviews, including Ng et al,^38^ who reported a forward citation search of the earliest identified included study, and Kirkland et al,^24^ who reported a forward citation search of a “sentinel paper”. Other reviews in this category did not report the specific studies that were used, including three that reported backward citation searching^42^, ^43^, ^44^ and three that reported forward citation searching.^28^, ^29^, ^39^

*Included studies* were used for backward citation searching in 94 of 159 (59%) reviews and forward citation searching in eight of 15 (53%) reviews that reported the set of studies used. Included studies are by convention listed in full in a Cochrane review.^19^

What we have labelled as *identified studies* were used for backward citation searching in 62 of 159 (39%) reviews that reported a set of studies and forward citation searching in two of 15 (13%) reviews that reported a set of studies. In seven reviews, our definition of identified was clearly apparent; ie, citation searching was conducted using studies excluded from the review (as well as included), or prior to agreeing inclusion/exclusion of studies.^45^, ^46^, ^47^, ^48^, ^49^, ^50^, ^51^ Dietrich et al^45^ and Huf et al^47^ reported conducting backward citation searching using included and excluded studies; MacDonald et al,^48^ Romano et al,^49^ and Wiysonge et al^51^ reported conducting backward citation using potentially eligible studies; and Howcroft et al^46^ and Walters et al^50^ reported conducting backward citation searching using studies retrieved for full‐text screening.

The majority of reviews in this category (n = 57) reported only that identified or retrieved studies were used for citation searching. For example, Di et al^52^ reported that “review authors searched the reference lists of identified studies,” Gregorio et al^53^ reported that they “checked the reference lists of all studies identified by the … [search] methods,” and Watson et al^54^ reported that the “reference lists of articles retrieved by electronic searches were searched for additional citations.” These examples imply that citation searching was conducted using every individual study identified. However, unless the number of studies identified overall was very low, this seems unlikely, and it may be more plausible that the review authors are using the term identified synonymously with included.

*Systematic reviews* were reported as used for backward citation searching in 65 of 159 (41%) reviews that reported a set of studies and forward citation searching in one of 15 (7%) reviews that reported a set of studies.

#### 3.2.2 What citation indexes are used and how frequently?

Of the 18 Cochrane reviews that reported using a citation index to conduct citation searching, 15 (83%) reported the name of the citation index(es) used. Google Scholar was reported in one review^55^; Scopus was reported in two reviews^24^, ^29^; and Web of Science was reported in 13 reviews,^26^, ^27^, ^28^, ^29^, ^31^, ^32^, ^34^, ^35^, ^36^, ^37^, ^38^, ^39^, ^40^ including seven reviews^26^, ^27^, ^28^, ^29^, ^32^, ^34^, ^39^ that reported searching the Science Citation Index (a subset of the Web of Science) in particular and one review^35^ that reported searching the Core Collection (which includes the Social Sciences Citation Index and Arts & Humanities Citation Index) in particular.

Van Mens et al^40^ reported conducting forward citation searching using PubMed (in addition to Web of Science), which probably refers to a Similar Articles search as PubMed does not facilitate citation searching. A Similar Articles search uses an algorithm to detect similar articles to a source study rather than identifying citing or cited articles.^56^ In addition to Van Mens et al,^40^ we serendipitously identified a small number of reviews that reported using Similar Articles search or equivalent searches in other databases.^29^, ^57^ However, because we were not systematically searching for this search method, it may have been reported more widely in the sample.

#### 3.2.3 The frequency of using a citation index to conduct backward citation searching

None of the reviews that reported backward citation searching reported using a citation index for this purpose. We have assumed that no mention of a citation index implied that backward citation searching was conducted manually. However, only three reviews explicitly reported using a manual approach, and in some of the remaining 169 reviews, authors might have failed to report the use of a citation index. In confirmation that the practice does exist, we identified one review that reported using Web of Science for backward citation searching in a previous iteration of the review; however, this was not repeated for the update review captured in our 3‐month cross section.^58^

#### 3.2.4 The frequency of using more than one citation index

Of the 15 reviews that reported the name of a citation index, one review (7%) reported using multiple citation indexes for forward citation searching, namely, Scopus and Web of Science.^29^ All other reviews that reported the use of a named citation index reported one citation index.

#### 3.2.5 Nonstandard approaches to citation searching, eg, snowballing

None of the reviews reported snowball searching nor did we detect any other nonstandard approaches other than the aforementioned Similar Articles search in PubMed.

### 3.3 Reporting of citation searching

Here, we present our findings on how citation searching was reported in the sample. Table 4 shows the number of Cochrane reviews that reported our proposed combination of MECIR standards and suggested details to report about backward and forward citation searching respectively and the detail required by the MECIR standards alone.^19^

**Table 4:** Detail reported about citation searching in cross section of Cochrane reviews

	Detail	BCS	FCS
		n = 172	n = 18
1	Name of citation index(es)/manual approach		
	Citation index(es)	0 (0)	16 (89)
	Manual (transparently reported)	3 (2)	‐
	Manual (assumed^a^ *)*	169 (98)	‐
2	Set of studies (transparently reported^b^)	94 (55)	10 (56)
3	Date searched	‐	2 (11)
	**All suggested details reported**	**1 (<1)**	**1 (6)**
	**All MECIR details reported**	**169 (98)**	**16 (89)**

*Note*. Numbers outside parentheses are totals, and numbers inside parentheses are percentages. All figures are calculated according to the total number of included reviews for backward (*n* = 172) and forward (*n* = 18) citation searching, respectively.

Bold = Sum Total.

Abbreviations: BCS, backward citation searching; FCS, forward citation searching.

a Manual approach assumed in absence of mention of citation index.

b Reviews that reported using included studies (which are listed in full in the review by convention) and studies that reported using key or other studies and have reported the specific studies.

Only two reviews reported all relevant details in our suggested checklist: one (less than 1% of total)^59^ for backward citation searching and one (6% of total)^34^ for forward citation searching. The number of reviews that fulfilled the requirements for the MECIR standards alone (ie, not including our suggested details to report) for both backward and forward citation searching was much higher (98% and 89%, respectively).^19^

Although no reviews reported using a citation index for backward citation searching, only three reviews (2%) explicitly stated that a manual approach was used.^38^, ^48^, ^59^ Reports of citation searching in some reviews used phrases suggestive of manual checking rather than a citation index, such as “we scanned the reference lists of relevant studies,” but we felt this was still not fully transparent. Hence, these are recorded as *manual (assumed)* in Table 4. Because there is no requirement to report how backward citation searching was conducted in the MECIR standards, these reviews are still fully MECIR compliant in terms of reporting.^19^ Only one review used the specific phrase *backward citation searching* to describe the search method,^25^ and all other reviews described this search method as *checking reference lists* or used similar phrases such as *inspecting references lists* or *examining reference lists*.

Just over half of reports of both backward (55%) and forward (56%) citation searching were fully transparent with respect to the set of studies used. The remaining reports detailed that either *key* or *identified* studies were used for citation searching without reporting the specific studies used in either case, or did not report a set of studies. As noted above, we acknowledge that some review authors might have used the word identified synonymously with included when describing the set of studies used.

The location of reports about citation searching in the sample of reviews is presented in Table 5. Almost all reviews reported citation searching in the methods section. A small number reported backward citation searching in the abstract or PRISMA flowchart without also mentioning in the main text.

**Table 5:** Location of reporting of citation searching in cross section of Cochrane reviews

Location^a^	BCS	FCS
	n = 172	n = 18
Abstract	9	0
Methods	162	18
PRISMA flowchart	1	0

Abbreviations: BCS, backward citation searching; FCS, forward citation searching.

a Reports in the abstract or PRISMA flowchart are only recorded if this was the sole location that citation searching was reported.

## 4 DISCUSSION

### 4.1 Conduct of citation searching

The conduct of backward citation searching in the cross section of Cochrane reviews appeared to be relatively uniform compared with forward citation searching. We have tentatively concluded that backward citation searching was conducted using a manual approach in all included reviews; however, this is dependent on the accuracy of our assumption that non‐reporting of a citation index implies that a manual approach was used. The option to conduct backward citation searching using a citation index and export the results to reference management software has the potential to facilitate a more systematic and transparent approach than manual checking, by allowing multiple screeners to code and compare the results of screening and easily share the results with interested third parties. Furthermore, de‐duplicating a large set of citations from multiple studies avoids the potential for screening the same cited study or studies multiple times. A shortcoming of this approach is the risk that cited studies are not indexed or not established as citations in the selected citation index. We suggest that searchers use their discretion as to whether to use a manual or citation index assisted approach.

The stipulation that included studies are used for backward citation searching is the only MECIR standard on the conduct of citation searching and is likely to account for the low number of reviews opting to conduct backward citation searching using key studies.

More variation in approach was found in the reviews that reported conducting forward citation searching. A manual approach is not an option when forward citation searching, but there are at least three available citation indexes that can be used. The popularity of Web of Science in the sample could simply be because it is the only available subscription‐based citation index in the review authors' institutional library holdings. Cochrane review authors might also value the option to easily search specifically science content in Web of Science via the Science Citation Index, as was evident in several reviews in the sample.

Google Scholar, despite being freely available, was the least popular citation index. This might be due to several shortcomings documented in the information science literature. For example, the relatively basic facilities for exporting results to reference management software can make the process cumbersome and time consuming.^60^ There is also increased incidence of duplicate citations due to its automated indexing of content.^61^ However, Google Scholar should not be dismissed as a useful tool, particularly for identifying grey literature, which has been estimated to comprise around half (48%‐65%) of Google Scholar's content, including theses/dissertations, books and book chapters, conference proceedings, preprints, and reports, most of which are not indexed in Scopus or Web of Science.^62^ This potentially makes Google Scholar particularly useful for identifying hard‐to‐find studies via citation searching, by combining a large amount of unique content with the aforementioned advantages of searching using citations.

The use of multiple citation indexes can avoid some of the shortcomings of using one citation index; however, this approach was only reported in one review. The time and resource implications of conducting multiple searches might be influential in the decision to use one citation index.

The relatively more frequent use of key studies for forward citation searching compared with backward citation searching is likely to be influenced by the lack of a MECIR standard stipulating the required set of studies.^20^ It might also be influenced by the suggestion in the Cochrane Handbook that citation indexes can be used for identifying citations of an important study.^2^ There is a risk that using key studies for citation searching introduces bias. Ultimately, however, citation searching in general is open to biases associated with citation practice, such as the selective citation of studies with positive results.^2^ Thus, studies with the same or similar research question are not always linked via citations.^12^

### 4.2 Reporting of citation searching

Compared with text‐based search methods such as searching bibliographic databases^63^ and web searching,^23^, ^64^ there is relatively little to report about citation searching in order to ensure transparency. We still, however, identified aspects of citation searching that could be better reported including the use of a manual approach for backward citation searching, the date of the search for forward citation searching, and the set of studies used for backward and forward citation searching. These details go beyond that required by the MECIR standards.^19^

We also suggest that it is optimal for review authors to report that citation searching was conducted in the methods section of the main body of the report, rather than only in the abstract or PRISMA flowchart, as witnessed in a small number of reviews in the sample. Reports that are not mentioned in the main text could be missed. Full details of citation searching, such as a list of studies used, can be reported in the appendices or supplementary material.

### 4.3 Comparison with other studies

Page et al present the reporting characteristics of a cross section of systematic reviews published in February 2014, including 45 Cochrane reviews.^10^ Of the 45 Cochrane reviews, 84% reported backward citation searching and 18% reported other search methods including forward citation searching.^10^ These findings are similar to the prevalence of backward (87%) and forward (9%) citation searching in our sample of Cochrane reviews. Horsley et al reviewed 12 studies that evaluated the effectiveness of backward citation searching as a supplementary search method for a systematic review.^3^ The frequency of using a manual approach or citation index is not reported, but they do report the set of studies used. A smaller proportion of systematic reviews than in our study used included studies (25% versus 59% in our review), and a proportion of systematic reviews only used other systematic reviews (33% versus 0% in our study). These differences could be explained by the inclusion of a wide variety of systematic reviews (ie not just Cochrane reviews) and the wider time frame in which the reviews in their sample were conducted (1985‐2005).^3^

### 4.4 Limitations

We have acknowledged that the descriptions of the set of studies used to conduct citation searching were in some cases ambiguous, particularly with respect to the difference between included studies and identified or retrieved studies. Although we categorized these descriptive accounts as two separate approaches, we acknowledge that the difference may be semantic rather than procedural. Thus, the number of reviews that actually used included studies might be higher than reported.

## 5 CONCLUSION

Our findings show variations in the conduct and reporting of citation searching. Some of the variations of conduct, such as the use of a particular citation index or the set of studies used, might simply reflect the available time or resources. However, particularly for forward citation searching, this might also reflect the need for more evidence‐based research and guidance on different approaches and more detailed methodological standards. Furthermore, we identified examples where citation searching could have been reported more transparently in fulfilment of the requirement for systematic reviews to include sufficient reporting for the methods to be reproducible. This goes beyond the requirements of the MECIR standards to include the approach used for backward citation searching, the date forward citation searching was conducted, and the sets of studies used for backward and forward citation searching.^19^

## CONFLICT OF INTEREST

The author reported no conflict of interest.

## AUTHOR CONTRIBUTIONS

S.B. was involved in all stages of the study. A.B. contributed to designing and piloting the data‐extraction form and read and commented on the final manuscript. M.R. contributed to designing and piloting the data‐extraction form and data extraction and read and commented on the final manuscript.

## Data Availability

The data that support the findings of this study are available from the corresponding author upon reasonable request.
